# Evaluation of Macular Thickness Changes Following Large Horizontal Rectus Muscle Recession: A Prospective Cohort Study

**DOI:** 10.7759/cureus.43145

**Published:** 2023-08-08

**Authors:** Konstantinos Paraskevopoulos, Christina Karakosta, Maria Liaskou, Georgios Feretzakis, Dimitrios Papakonstantinou, Konstantinos Droutsas, Ilias Georgalas

**Affiliations:** 1 Ophthalmolgy Department, Penteli General Hospital for Children, Athens, GRC; 2 First Ophthalmology Department, "G. Gennimatas" Hospital, National and Kapodistrian University of Athens, Athens, GRC; 3 Ophthalmology Department, Penteli General Hospital for Children, Athens, GRC; 4 School of Science and Technology, Hellenic Open University, Patras, GRC

**Keywords:** spectral domain optical coherent tomography, central macula thickness, oct macula, oct (optical coherence tomography), strabismus surgery

## Abstract

Aim

The aim of the study was to evaluate the long-term effect of large horizontal rectus muscle recession on macula thickness using spectral domain optical coherence tomography (SD-OCT).

Material and methods

Forty-two children were included in the study. The intervention groups were the medial rectus (MR) group (=20 eyes ) and the lateral rectus (LR) group (=22 eyes), including the eyes that underwent large medial and lateral rectus muscle recession, respectively. The control group included the fellow 42 unoperated eyes of the same children. Each eye was scanned using Topcon Maestro2 OCT-Angiography (OCTA; Topcon, Tokyo, Japan) preoperatively and then two months following surgery. A paired t-test was used to compare the mean difference in macular thickness between the intervention and control groups using the statistical program R (R Foundation for Statistical Computing, Vienna, Austria).

Results

The mean change in central, parafoveal, and perifoveal macular thickness of the intervention group was not statistically significant.

Conclusion

The long-term changes in macular thickness, as evaluated using SD-OCT both for the central and peripheral regions of the fovea, following large horizontal rectus muscle recession surgery, are not statistically significant.

## Introduction

Strabismus, characterized by misaligned eyes, is a prevalent condition among children worldwide, reaching 5% of preschool-aged children, and can lead to visual impairment if left untreated [1,2]. One common intervention for managing strabismus is horizontal rectus muscle recession, which involves detaching and repositioning the muscles responsible for eye movements to correct the misalignment [3]. This surgical intervention, while effective, has raised concerns about potential postoperative trauma and its long-term effects on the eye's macular region, the area responsible for central vision [4]. As macular damage can potentially lead to low quality of vision, understanding the impact of this surgery on macular thickness (MT) is of utmost importance [5].

Optical coherence tomography (OCT) has emerged as a reliable tool to measure MT non-invasively, thus providing valuable insights into any structural changes in the retina post-surgery [6,7]. Prior studies using OCT to evaluate MT after strabismus surgery have been largely inconclusive, with some studies suggesting a significant change while others report no substantial effect [8-11]. It is crucial to mention that those previous studies used an older-generation OCT.

However, these earlier studies often lack a robust control group for comparison and do not always specify the surgical techniques used. Moreover, many of these studies focus on short-term effects and fail to elucidate the long-term impacts on MT [12]. Our study aims to fill these gaps by comparing the long-term effects of 6 mm medial rectus (MR) and 9 mm lateral rectus (LR) recession surgeries on the MT of pediatric patients using automated, robotic-capture, high-resolution spectral domain OCT. These large muscle recessions lie in close proximity to the macula, which may be affected. To our knowledge, this is the first study to make a direct comparison between MR and LR recession, while using the fellow unoperated eyes as control.

## Materials and methods

The study was prospective, and the observation period lasted two months. The study was conducted in Penteli General Hospital for Children in association with the 1st Ophthalmology Department of "G. Gennimatas" Hospital of Athens, Greece.

The study protocol was approved by the Institutional Ethics Committee, and the study was conducted in accordance with the Declaration of Helsinki and informed consent was obtained from the guardians of all children included in the study. 

A total of 42 children, with a deviation of less than 20 prism diopters, were included in the study. The medial rectus muscle was operated on in 20 children and the lateral rectus muscle in 22 children. The children included in the study underwent uncomplicated large unilateral horizontal rectus muscle recession of one muscle only and the fellow eye served as control. All children included in the study had central fixation and best corrected visual acuity better than 20/25 in the deviating eye. Patients with a previous history of ocular surgery, trauma, sensory strabismus, maculopathy, or retinal disease, as well as patients who could not maintain reliable fixation or those who did not follow the scheduled visit after the surgery were excluded from the study.

Two main intervention groups were created, the medial rectus (MR) group, including the eyes that underwent 6 mm medial rectus muscle recession, and the lateral rectus (LR) group, including the eyes that underwent 9 mm lateral rectus muscle recession. The control group included the fellow unoperated eyes of the same children. All patients included in the study were examined preoperatively and then two months postoperatively. The preoperative ophthalmological examination included cycloplegic refraction, prism cover test, slit lamp examination, and fundoscopy. Each eye was scanned using the Topcon Maestro2 OCT-Angiography (OCTA; Topcon, Tokyo, Japan), preoperatively and then two months following surgery. OCTA Topcon Maestro2 is a robotic spectral domain OCT that automatically provides high-quality and high-resolution images.

Each OCT scan was centered on the fovea by providing a central, internal fixation mark. Central, parafoveal (superior, inferior, temporal, nasal), and perifoveal (superior, inferior, temporal, nasal) MT were measured. The central 1 mm diameter field of the ETDRS grid was defined as the central MT area. The parafoveal MT area refers to the pericentral ring with a 3-mm diameter while the perifoveal MT area refers to the pericentral ring with a 6-mm diameter.

**Figure 1:**
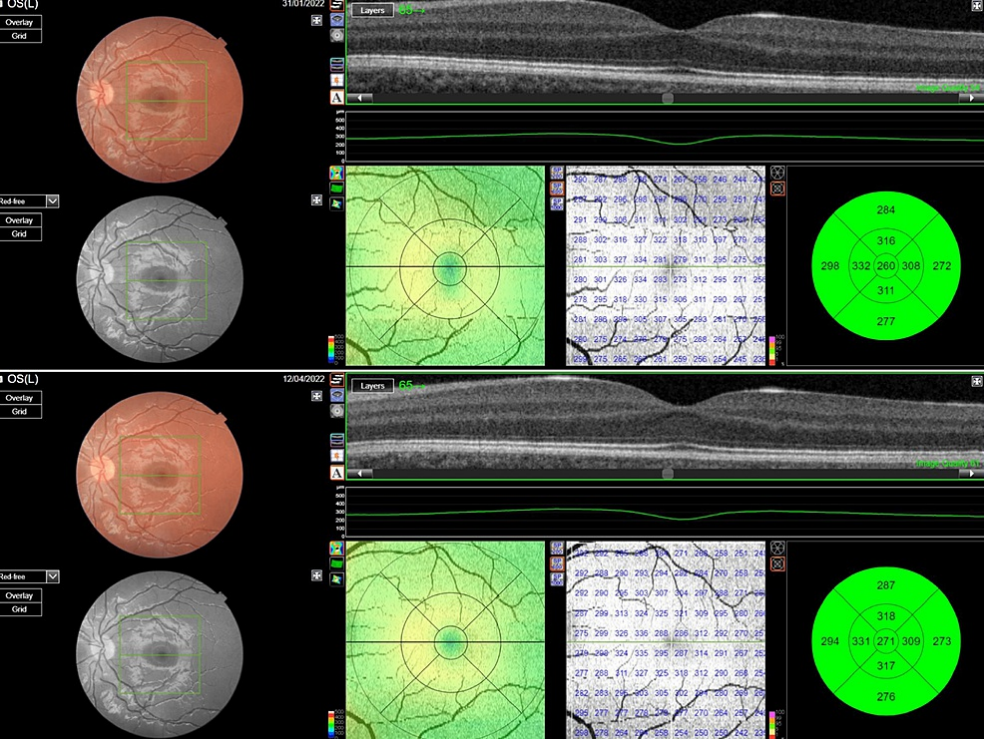
OCT scans of the left eye of a child who underwent medial rectus muscle recession Central MT is shown in the inner ring while parafoveal and perifoveal MT is shown in the middle and outer rings, respectively. The top image shows the preoperative measurements and the bottom image shows the postoperative ones.

OCT scans were repeated until good quality was obtained (image quality>60). Figure 1 presents an example of pre and postoperative OCT scans of a child, who underwent 6 mm medial rectus muscle recession of his left eye, showing the central, parafoveal, and perifoveal zones.

All surgical interventions were performed by the same ophthalmologist surgeon (name initials KP) under general anesthesia. All patients underwent horizontal rectus muscle recession surgery, applying the fornix approach technique [13]. In particular, a small incision was made through the conjunctiva and Tenon’s capsule, and a surgical plane was formed down to the bare sclera. A hook was passed parallel to the insertion of the muscle for muscle capture and isolation. A second muscle hook was then passed behind the first one to reassure that all muscle fibers were isolated. The muscle was carefully cleaned of all its fascial attachments and its insertion was exposed. A double-armed 5-0 absorbable polyglactin 910 (Vicryl, Ethicon, Raritan, New Jersey) suture with a speculated needle was passed superficially through the sclera, slightly anterior to parallel with the muscle insertion. The sutures were pulled to advance the muscle and then tied tightly. During the surgery, a hemostatic sponge of oxidized cellulose was used for hemostasis, when necessary, and no use of monopolar cutting diathermy was made.

Basic characteristics of the patients were summarized with means and standard deviations (SD) for normally distributed continuous variables or medians and interquartile ranges (IQR) for skewed data. The paired t-test was used to compare the mean difference of MT between the intervention and control groups (paired-eye comparison). A two-sided p-value of less than 0.05 was considered statistically significant. All analyses were carried out using the R programming language and the RStudio IDE.

## Results

A total of 42 children were included in the study, 28 boys and 14 girls. The mean age was 8.07 years, range=(5-16) in the MR group and 9.96 years, range=(4-16) in the LR group. Changes between preoperative and postoperative refractions of the patients are presented in a previous study by our team [8].

The mean central, parafoveal, and perifoveal MT measurements are given in Tables 1, 2. The mean change in central MT of the MR intervention group two months postoperatively was -1.50 μm (95% CI -15.798, 12.798) and was not statistically significant, with a p-value=0.828. The mean change in perifoveal MT was not statistically significant as well, with p-values of the superior, inferior, temporal, and nasal quadrants of 0.676, 0.738, 0.646, and 0.317, respectively). The mean change in central MT of the LR intervention group two months postoperatively was -1.810 μm (95% CI -15.798, 12.798) and was not statistically significant, with p-value=0.351. The mean change in perifoveal MT was not statistically significant as well, with p-values of the central superior, inferior, temporal, and nasal quadrants of 0.298, 0.497, 0.226, and 0.514, respectively. The mean change in central and parafoveal MT of the control group in the same observation period was not statistically significant as well.

**Table 1:** Macular thickness (MT) preoperatively (baseline) and two months postoperatively (postop) for the MR intervention group and control group A (fellow eyes of MR group) SD=Standard Deviation

MR intervention group	Baseline mean MT in μm (±SD)	Mean ΜΤ 2 months postop in μm (±SD)	Mean deviation	95% CI		p-value
Central	226.8±25.48	225.1±29.20	-1.50	-15.798	12.798	0.828
Superior parafoveal	313.0±15.41	312.4±12.06	1.25	-4.917	7.417	0.676
Inferior parafoveal	311.3±13.76	310.3±13.32	0.95	-4.923	6.823	0.738
Temporal parafoveal	300.2± 14.04	299.8±12.48	1.15	-4.020	6.320	0.646
Nasal parafoveal	310.9±17.86	308.1±16.73	2.90	-3.015	8.815	0.317
Superior perifoveal	283.5±12.21	283.9±10.18	0.50	-4.046	5.046	0.820
Inferior perifoveal	275.2±13.75	273.4±11.34	2.35	-1.616	6.316	0.230
Temporal perifoveal	267.9±11.86	267.1±10.56	1.45	-2.246	5.146	0.421
Nasal perifoveal	298.4±12.43	297.1±10.27	1.85	-3.209	6.909	0.453
Control group A						
Central	228.6±26.62	220.0±21.01	5.952	-3.841	15.746	0.219
Superior parafoveal	312.5±17.31	308.2± 13.72	3.285	-3.747	10.318	0.341
Inferior parafoveal	311.4±14.88	307.7±13.49	2.857	-3.048	8.762	0.324
Temporal parafoveal	300.4±14.09	297.6±14.38	1.952	-2.679	6.584	0.389
Nasal parafoveal	310.0±17.28	307.3±16.24	1.714	-3.476	6.904	0.498
Superior perifoveal	284.2±16.20	281.8±12.21	1.857	-3.681	7.395	0.492
Inferior perifoveal	273.7±12.59	273.2±13.14	-0.952	-6.556	4.651	0.726
Temporal perifoveal	268.4±12.71	266.4±12.37	1.476	-2.371	5.324	0.433
Nasal perifoveal	298.7±13.43	295.0±11.11	2.904	-2.372	8.181	0.264

**Table 2:** Macular thickness (MT) preoperatively (baseline) and two months postoperatively (postop) for the lateral rectus (LR) intervention group and control group B (fellow eyes of LR group) SD=Standard Deviation

LR intervention group	Baseline mean MT in μm (±SD)	Mean ΜΤ 2 months postop in μm (±SD)	Mean deviation	95% CI		p-value
Central	235.3±20.25	233.9±21.24	1.523	-1.810	4.858	0.351
Superior parafoveal	300.7±25.12	304.2±13.74	-5.333	-15.746	5.079	0.298
Inferior parafoveal	304.1±12.38	303.4±12.84	0.523	-1.057	2.104	0.497
Temporal parafoveal	293.9±11.54	292.8±11.75	0.952	-0.640	2.545	0.226
Nasal parafoveal	307.3±13.74	306.0±14.20	0.619	-1.325	2.563	0.514
Superior perifoveal	269.6±16.40	270.0±18.14	-0.761	-3.152	1.629	0.513
Inferior perifoveal	262.0±15.48	262.6±16.55	-0.476	-3.521	2.569	0.747
Temporal perifoveal	255.4±15.09	254.8±15.54	0.0476	-1.959	2.055	0.961
Nasal perifoveal	286.3±16.57	284.7±17.72	0.476	-0.931	1.883	0.488
Control group B						
Central	235.4±20.39	235.7±22.60	-0.619	-2.906	1.668	0.578
Superior parafoveal	306.9±12.72	305.1±14.32	1.047	-0.313	2.409	0.124
Inferior parafoveal	305.6±12.55	305.6±13.59	0.0476	-0.649	0.745	0.888
Temporal parafoveal	294.3±11.29	293.3±11.91	0.619	-0.664	1.902	0.326
Nasal parafoveal	308.7±13.82	307.9±14.39	0.809	-0.130	1.749	0.0874
Superior perifoveal	270.7±16.84	265.0±15.46	4.380	-0.701	9.463	0.087
Inferior perifoveal	262.1±16.09	263.9±16.18	-1.142	-2.883	0.598	0.186
Temporal perifoveal	255.2±15.08	253.7±15.82	1.190	-0.224	2.605	0.094
Nasal perifoveal	287.4±17.20	286.4±17.37	0	-0.801	0.801	1

The mean change in the perifoveal (superior, inferior, temporal, nasal) MT of the MR intervention group two months postoperatively was not statistically significant (p-values of peripheral superior, inferior, temporal, and nasal quadrant were 0.820, 0.230, 0.421, and 0.453, respectively). The mean change in the perifoveal (superior, inferior, temporal, nasal) MT of the LR intervention group two months postoperatively was not statistically significant (p-values of the central superior, inferior, temporal, and nasal quadrants were 0.513, 0.747, 0.961, and 0.488, respectively). The mean change in the perifoveal (superior, inferior, temporal, nasal) MT of the control group in the same observation period was not statistically significant as well. Pre and postoperative values of MT of the central, parafoveal, and perifoveal areas are presented in Tables 1, 2.

## Discussion

In this study, the potential long-term traumatic effect of large horizontal rectus muscle recession (6 mm of MR and 9 mm of LR) on MT using SD-OCT, was evaluated. The large muscle recession, particularly that of the lateral rectus, which is originally inserted at 7.7 mm from the limbus, lies in close proximity to the macula, which may be affected. Using a macular mapping test, this study evaluated the changes in MT. This type of surgery is commonly utilized in the correction of strabismus, a prevalent condition among children [14]. Despite its frequency of use, the potential long-term impacts of such procedures on macular thickness had remained unclear. The macula, especially the fovea in the center, is responsible for our central, high-resolution vision. Any potential changes to this region can have significant consequences for visual acuity [5]. Previous studies have reported conflicting results, with some suggesting significant changes in macular thickness following strabismus surgery, as a result of surgical trauma, traction for muscle isolation and separation, or the use of monopolar cutting diathermy, while other studies reported negligible impacts [8,15-17]. However, the present study provides strong evidence to suggest that such surgeries do not have a significant long-term impact on either central, parafoveal, or perifoveal MT. Nevertheless, it should be highlighted that in our study, no cautery was used and muscle handling was gentle.

One unique feature of the present study was the use of automated, robotic-capture, high-resolution, SD-OCTA Topcon Maestro2, which provided high-quality and high-resolution images. Another unique feature of this study was the use of fellow unoperated eyes as control. This allowed for comparison within the same individual, thereby effectively controlling for any potential confounders like age, systemic health, or genetic factors that might influence the macular thickness. Furthermore, the sample of this study was adequately sized and balanced in terms of gender, reducing the potential for bias.

No significant change was observed in either the central, parafoveal, or perifoveal MT in the eyes of children who underwent MR or LR recession surgery. These results were consistent across different age groups within our sample and were also consistent with the changes in refraction outlined in a previous study by our team [18].

The mean changes in different quadrants of the macula were also reported, namely, the superior, inferior, temporal, and nasal areas. Again, no significant changes were observed in these areas postoperatively, further confirming the lack of any major impact of the surgeries on macular thickness. In the present study, no changes in MT were observed in the two-month postoperative observation period, so it can be assumed that no further structural macular changes will take place linked to the surgery.

While these findings provide valuable clinical information, it is important to interpret them in light of certain limitations. In this study changes in macular perfusion, reflected by changes in the vascular density of the superficial and deep capillary plexus, were not evaluated due to the lack of cooperation of our young patients. Topcon Maestro2, being an SD-OCTA, has a visible broadband light, thus children may follow the light beam during scanning, leading to major difficulties in obtaining measurements. In contrast, swept-source OCTA, using a light source with a wavelength centered at 1050 nm, which cannot be seen by the human eye, and having a faster scanning speed, may overcome such problems.

Despite the limitations, the present study has significant implications for both clinicians and patients. The findings suggest that the horizontal rectus muscle recession surgery does not lead to any significant changes in macular thickness. This might allay fears of potential macular damage resulting from these surgeries, thus easing decision-making for both patients and surgeons.

## Conclusions

The principal objective of our research was to assess the potential effects of large horizontal rectus muscle recession, specifically 6 mm medial rectus (MR) and 9 mm lateral rectus (LR) surgeries, on MT, by using the Topcon Maestro2 OCTA. Given the prevalence of these procedures in pediatric ophthalmology for strabismus correction, understanding their impacts on ocular structures is crucial. The absence of change in MT applies both to the central and peripheral regions of the fovea, providing a strong indication that these surgeries do not have any structural effects on the macula.
